# The relationship between public risk preference and the underuse or overuse of preventive health services in the information age

**DOI:** 10.1016/j.pmedr.2024.102727

**Published:** 2024-04-12

**Authors:** Jiao Lu, Yanan Dong, Xiaoxiao Zhang, Yuan Wang, Zhongliang Zhou

**Affiliations:** a School of Public Policy and Administration, Xi’an Jiaotong University, Xi’an, Shaanxi, China; b School of Management, Shanxi Medical University, Taiyuan, Shanxi, China

**Keywords:** China, Risk preference, Information-acquisition ability, Preventive health services, Cancer screening, Information Age

## Abstract

The underuse or overuse of preventive health services by individuals is an outward behavioural reflection of their attitude towards disease risk, and they are strongly influenced by their information-acquisition ability. Therefore, we try to explore the relationship among the public risk preference, information-acquisition ability and underuse or overuse of preventive health services, in order to provide decision-making basis in the Information Age. The survey surveyed 2,211 respondents aged ≥ 18 in China from September to December 2019. Taking cancer screening as an example, the multiple price list (MPL) test and item response theory (IRT) model were used to measure individual risk preference and information-acquisition ability. The Logit model and Tobit model were used to estimate the relationship between risk preference, information-acquisition ability and underuse or overuse of preventive health services. Risk-seeking individuals were more likely to underuse preventive health services, while risk-averse individuals were more likely to overuse such services. Information-acquisition ability may improve the underuse of preventive health services in risk-seeking individuals but exacerbate the overuse of preventive health services in risk-averse individuals. Among the investigated information channels, the Internet is the most effective way for the public to obtain information. It is necessary to change the public’s incorrect perception of disease risks and risks associated with preventive health services. In the rapid development of the Information Age, improving public information-acquisition ability is a practicable way to correct the negative relationship between risk preference and individuals’ underuse or overuse of preventive health services.

## 1 Introduction

The rational use of preventive health services, such as immunization and disease screening, is one of the most effective ways to reduce incidence and mortality globally. While there is limited specific data to evaluate the effectiveness and cost-effectiveness of the use of preventive health services such as cancer screening in developing countries, including China (Chen et al., 2019), evidence from developed nations underscores their paramount importance for safeguarding public health and promoting well-being in resource-constrained developing countries.

Currently, China has established a comprehensive three-tier network of preventive health services based on disease prevention and control institutions and healthcare facilities at all levels. This network includes disease prevention and control centers, hospitals, and primary care institutions that recommend or provide 14 preventive services^1^ free of charge to the public as part of the National Basic Public Service Projects. However, there still exist several preventive health services that require individuals to bear the cost themselves, such as cancer screening and physical examinations. The cost of these out-of-pocket services varies with the level and technological advancements of institutions; for instance, cancer screening alone cost; between 0.81 % to 8.1 % of annual per capita income in 2022 (ranging from RMB300 to RMB3,000). However, due to the absence of detailed official guidelines on the utilization of preventive health services in China and institutional restrictions being lacking as well, individuals' perception biases may result in either underuse or overuse of preventive health services. Certain segments of the general population, who do not have health needs, exploit unnecessary preventive health services leading to their overuse (Korenstein et al., 2012, Brownlee et al., 2017), while individuals with actual health needs face challenges in effectively accessing preventive health services resulting in delayed treatment (Glasziou et al., 2017). These behaviors not only impose unwarranted economic burdens and physical harm on the public but also contribute to the wastage of medical resources and place a significant strain on national finances (Meester et al., 2015). Unfortunately, previous studies have primarily focused on exploring factors influencing the utilization behavior of preventive health services by the public (Hong et al., 2017, Kim et al., 2018), with relatively limited literature investigating the mechanisms underlying underuse or overuse.

Theoretically, when facing uncertain risks, individuals tend to make behavioural decisions based on their own psychological attitude towards risk, that is, risk preference (Kahneman and Tversky, 1979). The risk-seeking public may underestimate the likelihood of becoming ill and the severity of the disease, leading to underuse of preventive health services, while the risk-averse public tends to overstate the disease risks and blindly use preventive health services to avoid the risks (Leontine, 2017). However, there are relatively weak empirical studies that concentrate on the relationship between risk preference and the underuse or overuse of preventive health services. Therefore, we attempt to answer the questions: does the risk-seeking public tend to underuse preventive health services? Does the risk-averse public tend to overuse preventive health services?

In reality, we hope that everyone can be ‘rationalists’ who can correctly view the disease risk and rationally use preventive health services. People’s correct cognition of risks requires the support of information (Young et al., 2011). The widespread popularity of the internet and mobile terminals has greatly increased the public’s information accessibility and may influence their judgements of uncertain risk and its behavioural consequences by their information-acquisition ability (Wells et al., 2017). The stronger the public’s information-acquisition ability is, the more symmetrical the information is; furthermore, the more accurate the perception of their disease risk is, the more likely they are to redress individuals’ risk preference biases and reduce uncertainty in the use of preventive health services. Therefore, we attempt to answer the question: does information-acquisition ability moderate the relationship between the public’s risk preference and their underuse or overuse of preventive health services?

In summary, we measured individual risk preference and information-acquisition ability respectively, and used the Logit model and Tobit model to analyse the impact of risk preference on the underuse or overuse of preventive health services. Moreover, whether information-acquisition ability could correct individual risk preference and change their underuse or overuse of preventive health services was further discussed.

## 2 Materials and methods

### 2.1 Data/participants

A questionnaire-based cross-sectional study was performed to collect data in China from September to December 2019 using a multistage stratified random sampling method. A total of 96 communities (administrative villages) were included in the scope of investigation by using a random number table, and 28 members of the public aged ≥ 18 years were randomly selected from each community (administrative village) according to the right-handed principle [see Appendix A]. The study was approved by the Ethics Committee of Shanxi Medical University (Ref: 2018LL232), and all participants gave informed consent. All methods were performed in accordance with the relevant guidelines and regulations. A total of 2,688 questionnaires were collected, and 477 invalid questionnaires were eliminated because of omitting key information or filling in obvious errors. Finally, 2,211 valid questionnaires were obtained, and the effective recovery rate was 82.25 %.

### 2.2 Empirical models

#### 2.2.1 Dependent variables

There were two dependent variables in our study: individual underuse of preventive health services and individual overuse of preventive health services [see Appendix B].

#### 2.2.2 Independent variables

The core independent variables of this study were risk preference and information-acquisition ability.

We measured risk preference with reference to the study of Galizzi et al. in the context of healthcare to conduct MPL experiments (Table 1) (Galizzi et al., 2016) [see Appendix B]. If the public is likely to have cancer in the future, then it will develop in a very short time if no preventive measures are taken. Subjects were asked to choose between two prevention measures that extended their health (A and B). The payoffs in the prevention measures remain constant, but the probability associated with each payoff changes. A risk-neutral individual should switch from the ‘safe’ option (measure A) to the ‘risky’ option (measure B) only when the expected utility is greater in measure B than in A.

**Table 1:** Payoff matrix in HL MPL experimental test to determine risk preference of 2211 respondents aged ≥ 18 in China from September to December 2019.

**Pair**	**Measure A**				**Measure B**				**EV^A^**	**EV^B^**	**Difference**	**Range of CRRA**
	**p_1_**	**payoffs**	**p_2_**	**payoffs**	**p_1_**	**payoffs**	**p_2_**	**payoffs**				
1	0.1	200 days	0.9	160 days	0.1	385 days	0.9	10 days	164	48	117	r < -0.95
2	0.2	200 days	0.8	160 days	0.2	385 days	0.8	10 days	168	85	83	−0.95 < r < -0.49
3	0.3	200 days	0.7	160 days	0.3	385 days	0.7	10 days	173	123	50	−0.49 < r < -0.14
4	0.4	200 days	0.6	160 days	0.4	385 days	0.6	10 days	177	160	17	−0.14 < r < 0.15
5	0.5	200 days	0.5	160 days	0.5	385 days	0.5	10 days	182	198	−16	0.15 < r < 0.41
6	0.6	200 days	0.4	160 days	0.6	385 days	0.4	10 days	187	235	−48	0.41 < r < 0.68
7	0.7	200 days	0.3	160 days	0.7	385 days	0.3	10 days	192	273	−80	0.68 < r < 0.97
8	0.8	200 days	0.2	160 days	0.8	385 days	0.2	10 days	192	310	−118	0.97 < r < 1.37
9	0.9	200 days	0.1	160 days	0.9	385 days	0.1	10 days	197	348	−151	1.37 < r

We drew on Abdul-Salam and Phimister (2017) to construct an IRT model to accurately estimate the ability of individuals to acquire information. This method has been widely used in the fields of pedagogy, psychology and agricultural economic management to measure students’ reading ability, personal frustration level, degree of depression and farmers’ information-acquisition ability (Min and He, 2014, Ayis et al., 2018, Gao and Niu, 2019); however, there is relatively little empirical evidence available in the health field.

In terms of parameter estimation, we first took the individual’s use of the six information acquisition channels (internet, television, broadcast, preventive health institutions, newspapers and books, and being told by others) as the response variable to estimate its information-acquisition ability and adopted the 0–1 scoring method of the IRT model; that was, when an individual can use this channel to obtain information, the value was 1; otherwise, the value was 0. Thus, the item response matrix of individuals to the use of six information acquisition channels was constructed. Second, marginal maximum likelihood estimation (MMLE) was used to estimate the ‘discrimination’ and ‘difficulty’ parameters of the IRT model. Finally, Bayes expected a posteriori estimation (BEAPE) was used to estimate the information acquisition capability parameters in the IRT model based on the estimated ‘discrimination’ and ‘difficulty’ parameters.

#### 2.2.3 Control variables

Based on relevant studies, we considered eight control variables from two aspects of individual characteristics and family characteristics, including age, gender, education, participation in commercial medical insurance, chronic diseases, financial situation, family history of disease, and distance to the nearest preventive health institutions [see Appendix B].

### 2.3 Data analysis

We constructed two models of underuse and overuse of preventive health services. Among the 2,211 subjects in this study, 1,147 individuals (51.88 %) underused preventive health services, while only 144 individuals (6.51 %) overused preventive health services (Table 2). The variable of overused preventive health services contained a large number of zero values, which did not meet the normal distribution hypothesis; thus, it was a restricted dependent variable. Therefore, the binary discrete Logit model was used to construct the model of underuse of preventive health services, and the Tobit model was used to construct the model of overuse of preventive health services to correct biased results.

**Table 2:** Descriptive demographic data on 2211 respondents aged ≥ 18 in China from September to December 2019.

**Type**	**Variables**	**Assignments**	**N (%)**	**Mean ± SD**
Dependent variables	Underuse of preventive health services	1 = underuse, 0 = not underuse	1147(52) / 1064(48)	
	Overuse of preventive health services	1 = overuse, 0 = not overuse	144(7) / 2067(93)	
Independent variables	Risk preference (risk-seeking)	1 = risk-seeking, 0 = not risk-seeking	873(39) / 1338(61)	
	Risk preference (risk-averse)	1 = risk-averse, 0 = not risk-averse	1164(53) / 1047(47)	
	Information-acquisition ability	Information-acquisition ability		0.167 ± 0.04
Control variables	Age	actual age (years)		39.52 ± 15.68
	Sex	1 = male, 0 = female	1142(52) / 1069(48）	
	Education	actual years of education (years)		11.67 ± 3.27
	Participation in commercial health insurance	1 = purchase additional commercial health insurance, 0 = participating in the basic medical insurance only	361(16) / 1850(84)	
	Whether having chronic diseases	1 = yes, 0 = no	523(24) / 1688(76)	
	Financial situation	monthly average per capita household income (RMB)		3489.33 ± 2381.64
	Family history of disease	1 = with family history of disease, 0 = without family history of disease	188(9) / 2023(91)	
	Distance to the nearest preventive health institutions	distance from home to preventive health institutions (m)		1062.64 ± 422.54
Response variables	Obtain information via television	1 = yes, 0 = no	1758(79.51) / 453(20.49)	
	Obtain information via the Internet	1 = yes, 0 = no	1511(68.34) / 700(31.66)	
	Obtain information via broadcast	1 = yes, 0 = no	1430(64.68) / 781(35.32)	
	Obtain information via newspapers and books	1 = yes, 0 = no	1307(59.11) / 904(40.89)	
	Obtain information via preventive health institutions	1 = yes, 0 = no	1321(59.75) / 890(40.25)	
	Obtain information via being told by others	1 = yes, 0 = no	959(43.37) / 1252(56.63)	

## 3 Results

### 3.1 Descriptive analysis

Table 2 describes the distribution of risk preference and information-acquisition channels of the population. Table 3 shows a crosstab between risk preference and the use of different preventive health services.

**Table 3:** Frequency distribution characteristics between risk preference and underuse or overuse of preventive health services among 2211 respondents aged ≥ 18 in China from September to December 2019.

**Variables**	**normal**	**underuse**	**overuse**	**Total**
Risk preference (normal)	86	82	6	174
Risk preference (risk-seeking)	384	474	15	873
Risk preference (risk-averse)	450	591	123	1164
total	920	1147	144	2211

From an individual point of view, according to the interval distribution of the risk aversion coefficient of the respondents, 873 individuals (39.48 %) were in the interval [-0.5,0], and 1,164 individuals (52.65 %) were in the interval [0,1]. This indicates that the majority of the public is risk-averse. After considering the random errors, the average risk aversion coefficient of the public was 0. 981, and the whole sample was risk-averse. In summary, the distribution of individual risk preference of the public is consistent with the overall public risk preference with random errors accounted for, which indicates the reliability of the sample to a certain extent.

### 3.2 Estimated results of IRT model

#### 3.2.1 The estimated results of the item parameters

As shown in Table 4, the discrimination and difficulty parameters of the six information access channels passed the significance test at the 1 % level. This indicates that each of the preventive health services information access channels is closely related to the information-acquisition ability of individuals.

**Table 4:** Project parameter estimation results of the two-parameter logistic IRT model for the relationship between information-acquisition ability and information-acquisition channels among 2211 respondents aged ≥ 18 in China from September to December 2019.

**Channel**	**Discrimination**	**Standard error**	**Difficulty**	**Standard error**
Internet	2.503^***^	0.221	−0.571^***^	0.038
Television	2.099^***^	0.168	−1.064^***^	0.054
Broadcast	2.066^***^	0.159	−0.481^***^	0.039
Preventive health institutions	0.791^***^	0.072	−0.528^***^	0.073
Newspaper and books	−0.464^***^	0.065	0.894^***^	0.150
Being told by others	−1.137^***^	0.087	−0.291^***^	0.050

*Note*: *, ** and *** indicate 10%, 5%, and 1% significance levels, respectively.

#### 3.2.2 Estimated results of the information-acquisition ability parameter

We assumed that the parameter of the information-acquisition ability of individuals obeys the standard normal distribution. First, each individual can use at least two information acquisition channels and at most five simultaneously. Second, it is not the case that the more channels an individual can use, the greater its ability to acquire information. Third, when the number of channels available to individuals is the same, the greater the public can use the high discrimination parameter channels (such as the internet, television and radio), the greater the ability to acquire information.

In addition, 138 individuals (6.24 %) had information-acquisition ability parameters in the interval [0,0.5], and 359 individuals (16.24 %) were in the interval (0.5,1]. This indicates that only a small percentage of individuals have information-acquisition ability at an intermediate level. In addition, there were 1,120 individuals (0.66 %) whose information-acquisition ability parameter was less than 0 and 594 individuals (26.87 %) whose information-acquisition ability parameter was greater than 1. Overall, approximately half of the public has low information-acquisition ability, a quarter has intermediate information-acquisition ability, and a quarter has high information-acquisition ability.

### 3.3 Estimated results of the regression model

Before regression model analysis, we checked for multicollinearity of explanatory variables using variance inflation factors (VIFs) and obtained a mean VIF in our model of 1.56, and no VIF value larger than 4. Thus, multicollinearity is not an issue. First, this study examined the relationship between different risk preference and the underuse or overuse of preventive health services. The estimated results were shown in columns (1) and (2) in Table 5. Second, to further explore whether information-acquisition ability helps to modify the impact of individuals’ risk preference on the underuse or overuse of preventive health services, this study incorporated the interaction term of risk preference and information-acquisition ability into the model, and its estimated results were shown in the columns (3) and (4) in Table 5. The Wald values of the regression results were all large and reached the 1 % significance level, indicating a good fit of the model.

**Table 5:** Regression model estimation results of the relationship between underuse or overuse of preventive health services and risk preference and other factors among 2211 respondents aged ≥ 18 in China from September to December 2019, as well as the moderating effect of respondents' information acquisition ability on the relationship between insufficient or excessive use of preventive healthcare services and their risk preferences.

**Variables**	**Underuse^a^**	**Overuse^b^**	**Underuse^c^**	**Overuse^d^**
Risk preference (risk-seeking)	0.674*	−0.013	0.880**	−0.012
Risk preference (risk-averse)	−0.036	0.070***	−0.086	0.069***
Information-acquisition ability	−0.227***	0.005*	−0.079	−0.001
Risk preference (risk-seeking) × Information-acquisition ability			−0.543***	
Risk preference (risk-averse) × Information-acquisition ability				0.009*
Age	0.279***	−0.003***	0.290***	−0.003***
Sex	0.582***	−0.022**	0.511***	−0.023**
Education	−0.066**	0.003**	−0.059**	0.003**
Participation in commercial health insurance	−0.582**	0.036***	−0.590**	0.035***
Whether having chronic disease	0.846***	0.034***	0.876***	0.034***
Financial Status	0.000	0.000***	0.000	0.000***
Family history of disease	1.762***	0.044**	1.774***	0.045**
The distance to the nearest preventive health institutions	0.001***	0.000**	0.001***	0.000**
_cons	−12.326	0.055	−12.927	0.055
LR chi2	2244.63	208.74	2266.96	211.70
Prob > chi2	***	***	***	***
Pseudo R2	0.733	2.412	0.740	2.446

*Notes*: *, ** and *** indicate 10%, 5%, and 1% significance levels, respectively.

^a^ = the results of the Logit model with the underuse of preventive health services as a dependent variable;

^b^ = the results of the Tobit model with the overuse of preventive health services as a dependent variable;

^c^ = underuse of preventive health services as the dependent variable (Logit model), the variable of “risk preference” × “information-acquisition ability” was added;

^d^ = overuse of preventive health services as the dependent variable (Tobit model), the variable of “risk preference” × “information-acquisition ability” was added.

From the estimated results of (1) and (2), in the model of underuse of preventive health services, the estimated coefficient of risk-seeking was positive and passed the significance test (*p* = .057), indicating that risk-seeking individuals were more likely to have underused preventive health services compared to risk-averse individuals and risk-neutral individuals. In the model of overuse of preventive health services, the estimated coefficient of risk aversion was positive and passed the significance test (*p* = .000), indicating that risk-averse individuals were more likely to have overused preventive health services than risk-seeking individuals and risk-neutral individuals.

From the estimated results of (3) and (4), in the model of underuse and overuse of preventive health services, the estimated coefficient of the interaction term of risk-seeking and information-acquisition ability was negative, the estimated coefficient of the interaction term of risk-averse and information-acquisition ability was positive, and both passed the significance test (*p* = .000, *p* = .085). This suggests that information-acquisition ability improved the underuse of preventive health services in risk-seeking individuals, but further exacerbated the overuse of preventive health services in risk-averse individuals.

As shown in Table 5, in the model of underuse of preventive health services, age, gender, chronic diseases, family history of disease, and the distance to the nearest preventive health institutions were positively correlated with underuse of preventive health services; education and participation in commercial health insurance were significantly negatively associated with underuse of preventive health services. In the model of overuse of preventive health services, education, participation in commercial health insurance, chronic diseases, family history of disease, and the distance to the nearest preventive health institutions were significantly positively correlated with overuse of preventive health services; age and gender had significant negative associations with underuse of individual preventive health services. These results were in general similar to the findings of the existing articles. In addition, the financial situation did not significantly affect the underuse or overuse of preventive health services. The reasons may be as follows: first, the high-income groups in the sample have time costs and tend to neglect the use of preventive health services; second, different income groups may spend more of their income on improving their economic level and physical capital accumulation.

### 3.4 Robust tests

To test the robustness of the estimated results, the Probit model was used to construct a model of underuse of preventive health services, and a Binary Choice model in a Zero-inflated model was used to construct a model of overuse of preventive health services. The results (Table 6) show that the relationship direction and significance of the estimated results of risk-seeking and risk-averse, information-acquisition ability and interaction are consistent with the results in Table 5, which indicates that the estimated results of the model are robust.

**Table 6:** Regression model estimation results for robustness testing based on constructing a model of underuse of preventive health services using the Probit model and a binary selection model using the zero-inflation model to construct a model of overuse of preventive health services (N = 2211).

**Variables**	**Underuse^a^**	**Overuse^b^**	**Underuse^c^**	**Overuse^d^**
Risk preference (risk-seeking)	0.400**	−0.618	0.464**	−0.620
Risk preference (risk-averse)	−0.049	1.181***	−0.079	1.150***
Information-acquisition ability	−0.143***	0.000	−0.048	0.114
Risk preference (risk-seeking) × Information-acquisition ability			−0.294***	
Risk preference (risk-averse) × Information-acquisition ability				0.072
_cons	−5.957	−2.741	−6.295	−2.744
LR chi2	2215.70	69.67	2241.32	71.74
Prob > chi2	***	***	***	***
Pseudo R2	0.724	−	0.732	−

*Notes*: *, ** and *** indicate 10%, 5%, and 1% significance levels, respectively.

^a^ = the results of the Logit model with the underuse of preventive health services as a dependent variable;

^b^ = the results of the Tobit model with the overuse of preventive health services as a dependent variable;

^c^ = underuse of preventive health services as the dependent variable (Logit model), the variable of “risk preference” × “information-acquisition ability” was added;

^d^ = overuse of preventive health services as the dependent variable (Tobit model), the variable of “risk preference” × “information-acquisition ability” was added.

## 4 Discussion

Our findings suggest that risk preference may largely affect the individuals' rational utilization of preventive health services, and this may be due to their perception of disease risks (disease susceptibility and severity) and to the rational trade-offs between the benefits (reduced disease risks and probable outcomes) and losses (expenses, physical and psychological pain) of preventive health services (Guvenc et al., 2011), which is verified by the health belief model (HBM) and protective motivation theory (PMT) (Rosenstock, 1974, Rogers, 1975). For one thing, risk-seeking individuals have less perception of disease risks and prefer to have good luck to avoid disease losses so that they tend not to or seldom use preventive health services; while risk-averse individuals have a high perception of disease risks, which leads to excessive attention to disease risks and excessive use of preventive health services. For another thing, it is widely acknowledged that preventive health services such as cancer screening are associated with physical (screening pain, bleeding, infection, etc.) and psychological damage (embarrassing emotions); compared with the possible positive results of cancer screening and the feelings of embarrassment and pain during screening, it is more difficult for the public to accept the pain, death, and expensive treatment costs associated with future cancer (Wong et al., 2013). At this point, it is clear from PMT that individuals’ misperception of disease risks is magnified, inappropriate protective motivation is induced, and the differentiation of risk preference on the underuse or overuse of preventive health services is further implemented (Prentice-Dunn and Rogers, 1986). The excessive focus on disease risk reduction among risk-averse individuals results in an overutilization of preventive health services, indicating a heightened sense of protective motivation. Conversely, the lack of protective motivation among risk-seeking individuals inadvertently leads to an underutilization of preventive health services.

The theory of behavioral economics and the information-motivation-behavioral model posit that adequate information can facilitate individuals' accurate comprehension of disease risks, as well as the risks, benefits, and applicable conditions associated with preventive health services. This understanding can subsequently enhance their motivation to engage in protective behaviors and promote rational utilization of preventive health services (Fisher and Fisher, 1992, Li and Chen, 2020). However, individuals' behavior can be influenced by information only after they have perceived it (Greyson and Johnson, 2015), particularly in today's era of information explosion. Hence, the information-acquisition ability shows a moderating effect on the relationship between risk preference and under- or over-use of preventive health services in our study, improving the underuse of preventive health service behavior in risk-seeking individuals but exacerbating the overuse of preventive health services behavior in risk-averse individuals. This may be related to the type of information, influenced by the media's tendency to exaggerate facts, and individuals may be surrounded by rumors that overemphasize risk and its harms (El Sherif et al., 2018, Saab et al., 2018). Such information may enhance the lack of protective motivation of risk-seeking individuals, but may also further exaggerate the protective motivation of risk-averse individuals, and then further exaggerate their overuse of preventive health services behavior. It should therefore place more emphasis on the need of risk-averse individuals for objective information.

Moreover, the estimated results of the item parameters show that people prefer to obtain information through convenient, fast and interactive new media, and the internet is the most effective way. With the continuous implementation of the national strategy for cyber development, as of June 2021, China had 1.011 billion netizens, and its internet penetration had reached 71.6 %, providing a good foundation for the dissemination of information (China Internet Network Informations Center, 2021). Meantime, relevant departments vigorously promoted the accessibility of Internet applications for special groups such as the aged and the disabled and continuously narrowed the digital divide between various groups of people and the Internet (General Office of the State Council, 2021, The Ministry of Industry and Information Technology, 2021). The internet, with its timeliness, effectiveness, and convenience, has become the most important source of obtaining information for various members of the public. In comparison, although television and broadcast have problems with timeliness and interactivity, they are still the main channels for all kinds of public individuals to obtain information due to their high popularity, authority and convenient operation. Although preventive health institutions can directly provide information to the public face-to-face, they ignore the investigation of the real needs of the public and do not give full play to its strengths of two-way communication and providing personalized information, resulting in their role not being effectively used. Obviously, the timeliness and interactivity of information obtained from newspapers and books are poor and the communication between relatives and neighbours is aimed at mostly emotional communication (Otene et al., 2015, Wu and Liu, 2016); therefore, these two channels have the least effectiveness for the public to obtain information.

### 4.1 Strengths and limitations

This study made the following attempts. First, we used the MPL method to classify individuals’ risk preference for disease to try to fill the gaps in the preventive health services literatures. Second, we compensate for the insufficient attention to the overuse of preventive health services in the previous literatures. Third, we introduced the IRT model into the health field to rigorously estimate individual information-acquisition ability.

There were some limitations to this study. First, preventive health services are rich in content, including routine physical check-ups, prenatal care, immunization, cancer screening, rehabilitation care and so on. However, this study examined only the underuse or overuse of some cancer screening services by individuals, other preventive health services should be included in the analysis framework in future studies. Second, unobserved confounding factors may exist in this study, and it remains to be verified whether these factors will impact the variables and estimated results in this study. Third, the frequency of individual use of preventive health services relied on self-reporting and may be subject to recall bias. Fourth, the survey used a hypothetical nature of the risk elicitation procedure, which may bias the true risk preferences to some extent. Fifth, there is a limited representation of elderly individuals potentially leading to an underestimation of the extent of underuse or overuse issues. Moreover, considering the substantial prevalence of underutilization of preventive health services among elderly participants and the absence of age restrictions on preventive healthcare services in China, we do not encompass overuse defined as persistently receiving a preventive health service beyond the recommended cessation age. This may result in a slight deviation in assessing underutilization.

## 5 Conclusion and implications

The results of our study show that risk-seeking individuals are more likely to underuse preventive health services, and risk-averse individuals are more likely to overuse preventive health services. Information-acquisition ability could correct risk-seeking individuals’ underuse of preventive health services but not the risk-averse individuals’ overuse of preventive health services. Moreover, the internet is the most effective channel for individuals to obtain information, while preventive health institutions have not played their role in information dissemination.

Thus, it should strengthen the applicability of information dissemination channels, correct the objectivity of exaggerated information, and focus on enhancing the public’s information-acquisition ability to correct the public’s incorrect perception of disease risks and risks associated with preventive health services and its negative influences. For one thing, it is imperative to further enhance the role of the internet in disseminating information on preventive health services, by encouraging experts to generate and promote popular science accounts, videos, articles, etc., pertaining to disease risks and preventive health services on online platforms. Additionally, intensifying specialized supervision of online information dissemination can augment the efficacy of public access to professional knowledge regarding disease risks and preventive health services. For another thing, there is a pressing need for substantial innovation in the tracking function of preventive health institutions regarding customized personalized information. Proactive measures should be taken by these institutions to implement age-appropriate and barrier-free transformations in health management applications specifically designed for elderly individuals and those with disabilities. For instance, introducing a user-friendly interface mode that is simple to operate and incorporating various barrier-free functions such as one-touch operation and text input prompts can significantly enhance the information-acquisition capabilities of these key demographic groups. Furthermore, it is crucial to encourage community workers to form healthcare teams that stay updated on the self-care requirements of community residents, establish a regular follow-up reminder mechanism, and provide targeted and timely beneficial information to the public alongside personal and family health records. As an illustration, communities could periodically disseminate national cancer statistics along with knowledge about cancer prevention exclusively to families affected by this disease.

## 6 Ethics approval and consent to participate

The study was approved by the Ethics Committee of Shanxi Medical University (Ref: 2018LL232), and written informed consent was obtained from all the participants. All methods were carried out in accordance with relevant guidelines and regulations.

## Funding

This research was funded by the Humanity and Social Science Foundation of Ministry of Education of China (No. 22YJA630059), China Postdoctoral Science Foundation (No. 2022T150514), the Interdisciplinary Project of Basic Research Expenses of Xi'an Jiaotong University in 2023 (No. SK2023038), and National Natural Science Foundation of China (No. 71804101). The funders had no role in the design, data collection, analysis, interpretation of the data, and writing of this work.

## CRediT authorship contribution statement

**Jiao Lu:** Writing – review & editing, Writing – original draft, Methodology, Funding acquisition, Data curation. **Yanan Dong:** Writing – review & editing, Visualization, Validation, Formal analysis. **Xiaoxiao Zhang:** Writing – review & editing, Visualization, Validation. **Yuan Wang:** Writing – original draft, Investigation, Formal analysis. **Zhongliang Zhou:** Writing – review & editing, Supervision, Resources, Funding acquisition.

## Declaration of competing interest

The authors declare that they have no known competing financial interests or personal relationships that could have appeared to influence the work reported in this paper.

## Data Availability

Data will be made available on request.

## Footnotes

1 The 14 preventive health services provided by the National Basic Public Service Projects encompassing: 1) Establishing health records for residents; 2) Providing health education to residents; 3) Reporting and managing infectious diseases and sudden public health events; 4) Supervising and co-managing population and family planning for improved health outcomes; 5) Managing the healthcare of children aged 0–6; 6) Ensuring maternal and child health management; 7) Promoting elderly health management initiatives; 8) Facilitating traditional Chinese medicine-based healthcare management practices; 9) Implementing vaccination prevention programs effectively; 10) Managing chronic disease patients (including hypertensive patients and type II diabetic patients); 11) Overseeing serious mental disorder patient care programs; 12) Administering tuberculosis patient management strategies;13) Offering free provision of contraceptives; and 14) Enhancing promotion of health literacy.
