# Supplementary material for: The relationship between public risk preference and the underuse or overuse of preventive health services in the information age
Source: Prev Med Rep. 2024 Apr 12;41:102727. doi: 10.1016/j.pmedr.2024.102727 (PMC11021990; doi:10.1016/j.pmedr.2024.102727)
Supplement: Supplementary Data 1 [file mmc1.docx]

**The sampling procedures:**

According to the order of provincial-level administrative region, prefecture-level administrative region, county-level administrative region, township-level administrative region and community (administrative village), a total of 96 communities (administrative villages) were included in the scope of investigation by using a random number table.

In the first stage, the sampling unit was provincial-level administrative region (including province, autonomous region, municipality directly under the central government, and special administrative region). Taking the number of medical institutions in each region in 2018 as the indicator of regional medical development level, all provincial-level administrative regions in Mainland China are divided into high, medium and low levels (excluding Hong Kong, Macao and Taiwan region). Two provincial-level administrative regions were randomly selected from each level, and a total of six provincial-level administrative regions were selected.

In the second stage, the sampling unit was prefecture-level administrative region (including prefecture-level city, prefecture, autonomous prefecture, league). Two prefecture-level administrative regions were randomly selected from each provincial-level administrative region, and a total of 12 prefecture-level administrative regions were selected.

In the third stage, the sampling unit was county-level administrative region (including municipal district, county-level city, county, etc.). Two county-level administrative regions were randomly selected from each prefecture-level administrative region, and a total of 24 county-level administrative regions were selected.

In the fourth stage, the sampling unit was township-level administrative region (including sub-districts, towns, townships, etc.). Two township-level administrative regions were randomly selected from each county-level administrative region, and a total of 48 township-level administrative regions were selected.

In the fifth stage, the sampling unit was the community (administrative village). Two communities (administrative villages) were randomly selected from each township-level administrative region, and a total of 96 communities (administrative villages) were selected.

According to the order listed on the People’s Government and a professional website about postal code (https://www.youbianku.com/), all sampling units were numbered.

Considering the increasing prevalence of chronic diseases among younger individuals and acknowledging that 18 years old is widely recognized as the age at which people attain decision-making capacity, and avoid excessive sample homogeneity caused by a similar time and place, starting from the main gate of the community (village entrance) and in accordance with the right-handed principle, 28 members of the public aged ≥18 years were randomly selected from each community (administrative village).
