# Supplementary material for: The relationship between public risk preference and the underuse or overuse of preventive health services in the information age
Source: Prev Med Rep. 2024 Apr 12;41:102727. doi: 10.1016/j.pmedr.2024.102727 (PMC11021990; doi:10.1016/j.pmedr.2024.102727)
Supplement: Supplementary Data 2 [file mmc2.docx]

**Empirical models:**

**Dependent variables**

Preventive health services encompass a wide range of categories (including routine physical check-ups, prenatal care, immunization, cancer screening, rehabilitation care, etc.) (U.S. Preventive Services Task Force, 2021), and it is difficult to form a unified standard to evaluate the use of different preventive health services. In view of China’s cancer incidence and mortality ranking first in the world and the cancer mortality among urban and rural residents ranking first among all diseases in China (National Bureau of Statistics of China, 2019; Sung et al., 2021), on September 20, 2019, the National Health Commission of the People’s Republic of China (2021) and ten other government departments announced the ‘The Healthy China Action-Implementation Plan for Cancer Prevention and Control (2019-2022)’, which clearly listed cancer prevention and control as one of the important measures to achieve health for all, created clear regulations and long-term promotion for the improvement of cancer prevention and control capacity, improvement of prevention and control service system, early diagnosis and treatment screening, and guarantee of rescue and control system (National Health Commission of the People’s Republic of China, 2021). Among cancer prevention and control strategies, cancer screening is the most effective way to reduce the burden of cancer disease (Siegel et al., 2018). Therefore, this study combined ‘Recommendations for Screening and Prevention of Common Malignant Tumours in Residents (2020)’ published by the Shanghai Anticancer Association and Fudan University Shanghai Cancer Center and studied the corresponding screening behaviours of colorectal cancer, liver cancer, thyroid cancer, lymphoma, oesophageal cancer, pancreatic cancer, gallbladder cancer and bladder cancer (Chen et al., 2020). Due to the difference in recommended screening frequency between the general public and high-risk public, we first judged whether the public was a high-risk group according to the family history of cancer and then identified two dependent variables according to ‘Recommendations for Screening and Prevention of Common Malignant Tumours in Residents (2020)’ (Chen et al., 2020).

In the model of the underuse of preventive health services, the general public or high-risk public did not use, or did use any one or more cancer screening services less than the recommended screening frequency, which represented the underuse of preventive health services and took a value of 1. Otherwise, the value was 0. In the model of the overuse of preventive health services, the general public or high-risk public used any one or more cancer screening services more than the recommended screening frequency and thus represented the overuse of preventive health services for a value of 1. Otherwise, it was 0.

**Independent variables**

The core independent variables of this study were risk preference and information-acquisition ability.

***Risk preference***

Existing health-related studies mainly used three methods to distinguish the public’s risk preference. The first is to adopt health-related risk behaviour variables, such as smoking and insobriety, as proxy variables to measure individual risk preference (Anderson and Mellor, 2008). This method can only infer individuals’ risk preference indirectly through a series of observed health-related risk behaviours rather than directly measuring, which may easily lead to measurement bias. The second is to infer public risk preference through risk behaviours in other areas, such as stock market participation and risky asset choice (Cocco,2005; Chen, 2015). This method is not only separated from the health issue but also lacks the standard of quantification and conversion between different subject disciplines; it is difficult to ensure the measurement reliability of individuals’ risk preferences for health-related issues. The third is the multiple price list (MPL) based on Holt and Laury (2002) and adapted by Galizzi et al. (2016a). MPL compensates for the possible result bias of the first two risk preference inference methods. It is able to get straight to the point of the study by intuitively and rigorously classifying individuals as risk-seeking, risk-neutral and risk-averse. This method has been applied to distinguish differences in risk preference between doctors and patients in Athens, Greece (Galizzi et al., 2016b). Also, Zhu et al. (2019) used it to classify the risk preference of Chinese patients with cardiovascular disease over 60 years of age. Therefore, in this study, the method of Galizzi et al. was used to distinguish the risk preference of the Chinese public (Galizzi et al., 2016a).

MPL is a widely used method in behavioral economics to measure users' risk preferences. It presents users with a series of different benefit scheme combinations based on the required scenarios for investigation, each combination contains a safe scheme and a risky scheme (with a probability of loss), and the stable income of the safe scheme decreases sequentially, while the expected income of the risky scheme remains constant. By observing the user's decision-making habits when the stable income of the safe scheme drops to a certain level at which they abandon the safe scheme and turn to choose the risky scheme, their risk preferences can be indirectly inferred (Holt and Laury, 2002; Galizzi et al., 2016a).

The specific experimental process is as follows:

We assumed that risk preferences were elicited within the expected utility theory (EUT) framework and calculated individuals’ risk preferences based on the MPL experiment.

First, the health utility function perceived by individuals was constructed:

| $U\left( H \right)=\frac{x^{1-r}}{1-r}$ | Eq. (A.1) |
| --- | --- |

where $x$ was payoffs defined as days of full health, $r$ was the coefficient of constant relative risk aversion, and $U\left( H \right)$ represented the utility that a subject perceives from obtaining the payoffs $x$. The expected utility by a subject of a given measure $j=A, B$ was the utility of each outcome $k=1, 2$ in that lottery, weighted by the probability $p_{k}$ of the outcome:

| ${EU}_{i}=\sum_{k=1,2} p_{ki}*U\left( x_{ki} \right)$ | Eq. (A.2) |
| --- | --- |

Second, according to the individuals’ switching points from measure A to measure B in the MPL, using MATLAB software to construct the expected utility equation ${EU}_{A}={EU}_{B}$, we calculated the range of Arrow-Pratt CRRA (Zhou et al., 2012). Third, referring to the study of Tanaka et al. (2006), the midpoint of the range was taken as the estimated value of each individual’s risk aversion coefficient to accurately identify each individual’s risk preference. A negative estimated value indicates that the individual is risk-seeking. A positive estimated value indicates that the individual is risk averse.

To prove the reliability of the individual risk preference obtained by the above method, the stochastic error was set when comparing expected utilities, the maximum likelihood method was used for structural estimation, and the public risk preference was obtained. The econometric tools referred to the paper of Harrison (2008).

***Information-acquisition ability***

Most of the existing studies reflect individuals’ information-acquisition ability by the ‘number of information acquisition channels’ or a composite index weighted according to the relative effectiveness of different information acquisition channels (Esselaar et al., 2007; Yang et al., 2018). However, a simple summation of numbers does not reflect the difference in individual information-acquisition ability that ‘the number of information-acquisition channels is the same, but the combination of information acquisition channels is different’, while empowered summed composite index suffers from the problem of arbitrary weight assignment caused by a priori value judgements (Vandemoortele, 2013), which does not accurately reflect the information-acquisition ability of individuals.

Therefore, to avoid the above problems, based on item response theory (IRT), this study drew on Abdul-Salam and Phmister’s study (2017) to construct an IRT model to accurately estimate the ability of individuals to acquire information, which was reflected by the use of various information acquisition channels of preventive health services, and a two-parameter logistic IRT model was constructed:

| $\pi_{ij}=\frac{exp\left[ r_{j}\left( i{nformation}_{i}-b_{j} \right) \right]}{1+exp\left[ r_{j}\left( i{nformation}_{i}-b_{j} \right) \right]}$ | Eq. (A.3) |
| --- | --- |

In Eq. (3), $\pi_{ij}$ was the probability that individual $i$ could obtain preventive health services information from channel $j$. The main channels for individuals to obtain preventive health services information were the internet (e.g., searching for information on computers and mobile phones), television, broadcast (e.g., radio, car broadcasts), preventive health institutions (e.g., publicity boards, lectures, and word of mouth by doctors or other relevant personnel), newspapers and books (e.g., buying your own paper materials), and being told by others (e.g., relatives, friends and colleagues), $i{nformation}_{i}$ was the parameter of individual $i$’s information-acquisition ability, and it was assumed that it followed the standard normal distribution. The larger the value is, the stronger the individual’s information-acquisition ability is. $r_{j}$ and $b_{j}$ represent the ‘discrimination’ parameter and ‘difficulty’ parameter of channel $j$, respectively. The larger $r_{j}$ is, the stronger the effectiveness of channel $j$ to help individuals obtain information is. The larger $b_{j}$ is, the more difficult it is for individuals to obtain information from channel $j$.

**Control variables**

Based on relevant studies, we considered eight control variables from two aspects of individual characteristics and family characteristics, including age, gender, education, participation in commercial medical insurance, chronic diseases, financial situation, family history of disease, and distance to the nearest preventive health institutions.

(1) Age (actual age). Young and middle-aged individuals may have a strong willingness to use preventive health services and be more prone to overuse preventive health services than other age groups (Ofoli et al., 2020).

(2) Gender (1 = male, 0 = female). Females would likely to pay more attention to the health status of themselves and their families and to have knowledge of the components of preventive healthcare compared to males (Ofoli et al., 2020).

(3) Education (actual years of education). Individuals with low education levels may either underuse or blindly pursue preventive health services (Fetterolf, 1999).

(4) Participation in commercial health insurance (1 = purchasing additional commercial health insurance, 0 = participating in basic medical insurance only). With the increasing awareness of preventive healthcare, an increasing number of the public are buying commercial health insurance for themselves and their families to enhance their ability to withstand health risks. As a strong complement to the basic health insurance system, commercial health insurance covers the cost of preventive care as much as possible, which may lead to unnecessary use of preventive health services by individuals (Farrow, 2010; Zhou et al., 2014; Hu et al., 2015). At present, the goal of full coverage of the basic medical insurance system has been essentially achieved, and the sample public in this study has all participated in basic medical insurance.

(5) Whether patients have chronic diseases (1 = yes, 0 = no). Minimally invasive preventive health services, such as cancer screening, are more likely to cause additional psychological burden and physical discomfort to patients with chronic diseases; therefore, they tend to underuse preventive health services (Batty et al., 2015). However, for the consideration of early detection and treatment, the public in the state of health or the incubation period of disease may increase the use frequency of preventive health services, resulting in overuse behaviour.

(6) Financial situation (monthly average per capita household income). The more affluent a household is, the more it spends on preventive health services, and the more prone it is to overuse preventive health services (Chen, 2015).

(7) Family history of disease (1 = with family history of disease, 0 = without family history of disease). The illness of family members may induce individuals to pay attention to their own health conditions, incline them to increase the frequency of use of preventive health services and lead to overuse behaviour.

(8) Distance to the nearest preventive health institutions (distance from home to preventive health institutions). The farther the distance to the nearest preventive health institutions, the more likely the public is to underuse preventive health services (Ci et al., 2020).

**References**

1. Abdul-Salam Y, Phimister E. Efficiency effects of access to information on small-scale agriculture: empirical evidence from uganda using stochastic frontier and IRT models. Journal of Agricultural Economics. 2017;68(2):494-517. doi:10.1111/1477-9552.12194.
2. Anderson LR, Mellor JM. Predicting health behaviors with an experimental measure of risk preference. J Health Econ. 2008;27(5):1260-74. doi:10.1016/j.jhealeco.2008.05.011.
3. Batty CA, Cauchi M, Lourenço C, Hunter JO, Turner C. Use of the analysis of the volatile faecal metabolome in screening for colorectal cancer. PLOS ONE. 2015;10(6):e0130301. doi:10.1371/journal.pone.0130301.
4. Chen L. The Utilization of preventive care services and its relationship with health status. Southeast University. 2015. doi:10.7666/d.Y2920103. (In Chinese)
5. Chen Z, Ye D, Zheng Y, Ni M. Recommendations for Screening and Prevention of Common Malignant Tumours in Residents (2020). Shanghai Anticancer Association and Fudan University Shanghai Cancer Center Press; 2020. (In Chinese)
6. Ci H, Ding X, Qiu D, Wu B. Studying on the utilization of preventive health care services for the housebound elderly from the perspective of social stratification. Chinese Health Service Management. 2020;37(9):713-7. (In Chinese)
7. Cocco JF. Portfolio Choice in the Presence of Housing. Review of Financial Studies. 2005;18(2):535-67. doi:10.1093/rfs/hhi006.
8. Esselaar S, Stork C, Ndiwalana A, Deen-Swarray M. ICT usage and its impact on profitability of SMEs in 13 African countries. Information Technologies and International Development. 2007;4(1):87-100. doi:10.1162/itid.2007.4.1.87.
9. Farrow FL. Overutilization and underutilization of preventive services in elderly populations, a conundrum. Marquette Elders Advisor. 2010;12(1):Article 6
10. Fetterolf DE. A Framework for Evaluating Underutilization of Health Care Services. American Journal of Medical Quality. 1999;14(2):89-97. doi:10.1171/106286069901400204.
11. Galizzi MM, Miraldo M, Stavropoulou C. In sickness but not in wealth: field evidence on patients’ risk preferences in financial and health domains. Medical Decision Making. 2016a;36:503-17. doi:10.1177/0272989X15626406.
12. Galizzi MM, Miraldo M, Stavropoulou C, van der Pol M. Doctor-patient differences in risk and time preferences: A field experiment. Journal of Health Economics. 2016b;50:171-82. doi:10.1016/j.jhealeco.2016.10.001.
13. Harrison. Maximum likelihood estimation of utility functions using stata. University of Central Florida. 2008. http://faculty.cbpp.uaa.alaska.edu/jalevy/protected/HarrisonSTATML.pdf. Accessed 1 October 2021.
14. Holt CA, Laury SK. Risk aversion and incentive effects. American Economic Review. 2002;92(5):1644-55. doi:10.1257/000282802762024700.
15. Hu H, Luan W, Li J. Medical insurance, health services utilization and excessive demands for medical services-the impact of medical insurance on utilization of health service of the elderly. Journal of Shanxi University of Finance and Economics. 2015;37(5):14-24. doi:10.13781/j.cnki.1007-9556.2015.05.002. (In Chinese)
16. National Bureau of Statistics of China. Health Statistical Yearbook 2019. CN: China Statistics Press; 2019. (In Chinese)
17. National Health Commission of the People’s Republic of China. The Healthy China Action-Implementation Plan for Cancer Prevention and Control (2019-2022). http://www.nhc.gov.cn/jkj/s5878/201909/2cb5dfb5d4f84f8881897e232b376b60.shtml. Accessed 1 October 2021. (In Chinese)
18. Ofoli JNT, Ashau-Oladipo T, Hati SS, Ati Lile, Ede Victor. Preventive healthcare uptake in private hospitals in Nigeria: a cross-sectional survey (Nisa premier hospital). BMC Health Serv Res. 2020;20(1):273. doi:10.1186/s12913-020-05117-5.
19. Siegel RL, Miller KD, Jemal A. Cancer statistics, 2018. CA: A Cancer Journal for Clinicians. 2018;68(1):7-30. doi:10.3322/caac.21442.
20. Sung H, Ferlay J, Siegel RL, Laversanne M, Soerjomataram I, Jemal A, et al. Global Cancer Statistics 2020: GLOBOCAN Estimates of Incidence and Mortality Worldwide for 36 Cancers in 185 Countries. CA Cancer J Clin. 2021;71(3):209-49. doi:10.3322/caac.21660.
21. Tanaka T, Camerer CF, Nguyen Q. Preferences, poverty and politics: experimental and survey data from vietnam. California institute of Technology. 2006. https://www.researchgate.net/publication/4820801. Accessed 1 October 2021.
22. U.S. Preventive Services Task Force. USPSTF A and B recommendations by date.https://www.uspreventiveservicestaskforce.org/Page/Name/uspstf-a-and-b-recommendations-by-date/. Accessed 1 October 2021.
23. Vandemoortele M. Measuring household wealth with latent trait modelling: an application to malawian DHS data. Social Indicators Research. 2013;118(2):877-91. doi:10.1007/s11205-013-0447-z.
24. Yang N, Zhou J, Ma L, Tang L. The impact of information access media on occupational choice of rural inhabitants—based on CGSS2013 survey date. Journal of Agrotechnical Economics. 2018;(05):52-65. doi:10.13246/j.cnki.jae.2018.05.005. (In Chinese)
25. Zhou Y, Zuo C, Chen Y, Lian H, Ye A. An experimental study of risk aversion in individuals with social preferences. Management World. 2012;(06):86-95. doi:10.19744/j.cnki.11-1235/f.2012.06.008. (In Chinese)
26. Zhou Z, Zhu L, Zhou Z, Li Z, Gao J, Chen G. The effects of China’s urban basic medical insurance schemes on the equity of health service utilisation: evidence from Shaanxi Province. International Journal for Equity in Health. 2014;13(1):23. doi:10.1186/1475-9576-12-23.
27. Zhu J, Shi Y, Li J, Zhang Z. Role of risk attitude and time preference in preventive aspirin use adherence. Journal of Evaluation in Clinical Practice. 2019;26(3):1-7. doi:10.1111/jep.13274.
